# Malaria prevalence among pregnant women in two districts with differing endemicity in Chhattisgarh, India

**DOI:** 10.1186/1475-2875-11-274

**Published:** 2012-08-10

**Authors:** Neeru Singh, Mrigendra P Singh, Blair J Wylie, Mobassir Hussain, Yeboah A Kojo, Chander Shekhar, Lora Sabin, Meghna Desai, V Udhayakumar, Davidson H Hamer

**Affiliations:** 1 Regional Medical Research Centre for Tribals, Jabalpur, Madhya Pradesh, India; 2 National Institute of Malaria Research Field Station, Jabalpur, Madhya Pradesh, India; 3 Division of Maternal-Fetal Medicine, Department of Obstetrics and Gynecology, Massachusetts General Hospital, Boston, MA, USA; 4 Center for Global Health and Development, Boston University, Boston, MA, USA; 5 Department of International Health, Boston University School of Public Health, Boston, MA, USA; 6 Department of Reproductive Health and Nutrition, Indian Council of Medical Research, New Delhi, India; 7 Malaria Branch, Division of Parasitic Diseases and Malaria, Center for Global Health, Centers for Disease Control and Prevention, Atlanta, GA, USA; 8 Section of Infectious Diseases, Department of Medicine, Boston University School of Medicine, Boston, MA, USA; 9 Zambia Centre for Applied Health Research and Development, Lusaka, Zambia

**Keywords:** Pregnancy, Malaria, India, *Plasmodium falciparum*, *Plasmodium vivax*, Anaemia, Low birth weight

## Abstract

**Background:**

In India, malaria is not uniformly distributed. Chhattisgarh is a highly malarious state where both *Plasmodium falciparum* and *Plasmodium vivax* are prevalent with a preponderance of *P. falciparum*. Malaria in pregnancy (MIP), especially when caused by *P. falciparum*, poses substantial risk to the mother and foetus by increasing the risk of foetal death, prematurity, low birth weight (LBW), and maternal anaemia. These risks vary between areas with stable and unstable transmission. The specific objectives of this study were to determine the prevalence of malaria, its association with maternal and birth outcomes, and use of anti-malarial preventive measures for development of evidence based interventions to reduce the burden of MIP.

**Methods:**

A cross-sectional study of pregnant women presenting to antenatal clinics (ANC) or delivery units (DU), or hospitalized for non-obstetric illness was conducted over 12 months in high (Bastar) and low (Rajnandgaon) transmission districts in Chhattisgarh state. Intensity of transmission was defined on the basis of slide positivity rates with a high proportion due to *P. falciparum*. In each district, a rural and an urban health facility was selected.

**Results:**

Prevalence of peripheral parasitaemia was low: 1.3% (35/2696) among women at ANCs and 1.9% at DUs (19/1025). Peripheral parasitaemia was significantly more common in Bastar (2.8%) than in Rajnandgaon (0.1%) (p < 0.0001). On multivariate analysis of ANC participants, residence in Bastar district (stable malaria transmission) was strongly associated with peripheral parasitaemia (adjusted OR [aOR] 43.4; 95% CI, 5.6-335.2). Additional covariates associated with parasitaemia were moderate anaemia (aOR 3.7; 95% CI 1.8-7.7), fever within the past week (aOR 3.2; 95% CI 1.2-8.6), and lack of formal education (aOR 4.6; 95% CI 2.0-10.7). Similarly, analysis of DU participants revealed that moderate anaemia (aOR 2.5; 95% CI 1.1-5.4) and fever within the past week (aOR 5.8; 95% CI 2.4-13.9) were strongly associated with peripheral and/or placental parasitaemia. Malaria-related admissions were more frequent among pregnant women in Bastar, the district with greater malaria prevalence (51% vs. 11%, p < 0.0001).

**Conclusions:**

Given the overall low prevalence of malaria, a strategy of enhanced anti-vector measures coupled with intermittent screening and targeted treatment during pregnancy should be considered for preventing malaria-associated morbidity in central India.

## Background

India contributes over one fifth (22.6%) of clinical episodes of *Plasmodium falciparum* and 42% of episodes of *Plasmodium vivax* globally [1,2]. An estimated 200,000 persons below 70 years of age die annually due to malaria in India [3]. Five states: Orissa, Chhattisgarh, Madhya Pradesh, Jharkhand, and West Bengal, are responsible for nearly two-thirds of malarial episodes and the majority of malarial deaths in India [3,4]. Past research has suggested a higher malaria prevalence among tribal populations or indigenous people [5-7].

**Table 1:** Baseline characteristics of pregnant women attending antenatal clinics and delivery units

	**ANC**		**DU**	
**Characteristic**	**Rajnandgaon n = 1498**	**Bastar n = 1198**	**Rajnandgaon n = 547**	**Bastar n = 481**
	**n**^**†**^**(%)**	**n**^**†**^**(%)**	**n**^**†**^**(%) or mean (± SD)**	**n**^**†**^**(%) or mean (± SD)**
Age (years)				
< 20	131 (8.7)	111 (9.3)	36 (6.6)	32 (6.7)
20-34	1325 (88.5)	1065 (88.9)	497 (90.9)	438 (91.5)
≥ 35	42 (2.8)	22 (1.8)	14 (2.6)	9 (1.9)
Prior pregnancies^1^				
Primigravid	600 (40.1)	631 (52.7)	236 (43.1)	270 (56.1)
Secundigravid	498 (33.2)	327 (27.3)	193 (35.3)	108 (22.5)
Multigravid*	400 (26.7)	240 (20.0)	118 (21.6)	103 (21.4)
Estimated gestational age at enrollment (weeks)**^,1^			37.8 (± 1.2)	35.8 (± 1.7)
< 20 weeks	587 (39.2)	455 (38.0)		
20-36 weeks	858 (57.3)	733 (61.2)		
≥ 37 weeks	53 (3.5)	10 (0.8)		
Caste^1^				
Scheduled caste	333 (22.3)	354 (29.6)	112 (20.6)	145 (30.2)
General caste	229 (15.3)	182 (15.2)	99 (18.2)	37 (7.7)
Other backward caste	767 (51.3)	226 (18.9)	270 (49.5)	73 (15.2)
Scheduled tribe	166 (11.1)	436 (36.4)	64 (11.7)	226 (47.0)
Education^1^				
No formal schooling	228 (15.2)	343 (28.6)	81 (14.8)	191 (39.7)
Primary (1-5 years)	287 (19.2)	330 (27.6)	115 (21.0)	157 (32.6)
Secondary (6-10 years)	760 (50.7)	380 (31.7)	258 (47.2)	107 (22.2)
Higher (> 10 years)	223 (14.9)	145 (12.1)	93 (17.0)	26 (5.4)
Socioeconomic characteristics				
Owns TV^1^	960 (64.1)	453 (37.8)	324(59.2)	135(28.1)
Owns bicycle	1113 (74.3)	893 (74.5)	436 (79.7)	358 (74.4)
Owns house^1^	1219 (81.4)	1129 (94.2)	446 (81.5)	475 (98.8)
Owns refrigerator	145 (9.7)	126 (10.5)	53 (9.7)	19 (4.0)
Roof material^1^				
Mud tiles	1059 (70.7)	1041 (86.9)	384 (70.2)	441 (91.7)
Corrugated iron/asbestos sheet	42 (2.8)	4 (0.3)	8 (1.5)	3 (0.6)
Cement/concrete	369 (24.6)	146 (12.2)	128 (23.4)	23 (4.8)
Other (non-permanent materials)	28 (1.9)	7 (0.58)	27 (4.9)	14 (2.9)
Wall material^1^				
Mud/sand/dung	719 (48.0)	473 (39.5)	271 (49.5)	207 (43.0)
Mud bricks	274 (18.3)	476 (39.7)	101 (18.5)	207 (43.0)
Cement bricks	494 (33.0)	232 (19.4)	164 (30.0)	58 (12.1)
Other (e.g., wood planks, grass & bamboo)	11 (0.7)	17 (1.4)	11 (2.0)	9 (1.9)
Primary cooking fuel^1^				
Wood	1030 (68.8)	1004 (83.8)	369 (67.5)	437 (90.9)
Charcoal	78 (5.2)	5 (0.4)	21 (3.8)	1 (0.2)
Gas	347 (23.2)	172 (14.4)	134 (24.5)	36 (7.5)
Other (kerosene & electric heater)	43 (2.9)	17 (1.4)	23 (4.2)	7 (1.5)

^†^ Numbers may not add to sample size secondary to missing data.

SD = standard deviation.

* Defined as ≥ 3 pregnancies.

** Gestational age calculated by fundal height in ANC and by Ballard score in DU subjects. Fundal heights measured when fundus was above the umbilicus. Unrecorded fundal heights considered a gestational age <20 weeks. Infants that were stillborn did not have a Ballard examination performed.

^1^p < 0.001.

**Table 2:** Use of malaria prevention measures by pregnant women attending antenatal clinics and delivery units

	**Antenatal clinics**		**Delivery units**	
**Prevention measures utilized**	**Rajnandgaon n = 1498**	**Bastar n = 1198**	**Rajnandgaon n = 547**	**Bastar n = 481**
Bed net in household*	556 (37.1)	541 (45.2)	166 (30.4)	147 (30.6)
Among bed net owners: ITN	7 (1.3)	2 (0.4)	2 (1.2)	3 (2.0)
Sleeps under net most nights*^†^	312 (56.1)	345 (63.8)	70 (42.2)	80 (54.4)
Slept under net last night*^†^	347 (62.4)	395 (73.0)	103 (62.0)	108 (73.5)
Taken malaria prophylaxis in pregnancy*	0	4 (0.3)	0 (0)	3 (0.6)
IRS of home*^†^	488 (32.6)	209 (17.5)	167 (30.6)	103 (21.4)

ITN = insecticide-treated bed net. IRS = indoor residual spraying.

Numbers presented are n (%). Numbers may not add to sample size secondary to missing data.

* p < 0.05 comparing Rajnandgoan with Bastar districts for ANC participants.

^†^ p < 0.05 comparing Rajnandgoan with Bastar districts for DU participants.

**Table 3:** Parasitaemia and anaemia among pregnant women attending antenatal clinics and delivery units

	**Antenatal clinics**	
	**Rajnandgoan n = 1498**	**Bastar n = 1198**
Peripheral parasitaemia^†^		
Overall*	1 (0.1)	34 (2.8)
By species*		
*P. falciparum*	0 (0)	29 (2.4)
*P. vivax*	1 (0.1)	5 (0.4)
Mixed	0 (0)	1 (0.1)
Anaemia		
Overall* (Hgb < 11 g/dL)	957 (63.9)	861 (71.9)
Moderate/severe* (Hgb < 9 g/dL)	183 (12.2)	249 (20.8)
	**Delivery units**	
	**Rajnandgoan n = 547**	**Bastar n = 481**
Peripheral parasitaemia^†^		
Overall*	3 (0.6)	16 (3.4)
By species*		
*P. falciparum*	1 (0.2)	12 (2.7)
*P. vivax*	2 (0.4)	3 (0.6)
Mixed	0 (0)	1 (0.2)
Anaemia		
Overall* (Hgb < 11 g/dL)	459 (83.9)	304 (63.9)
Moderate/severe* (Hgb < 9 g/dL)	64 (11.7)	91 (19.1)
Placental parasitaemia^†^		
Overall	16 (3.2)	17 (3.6)
By species		
*P. falciparum*	6 (1.2)	12 (2.5)
*P. vivax*	9 (1.8)	3 (0.6)
Mixed	1 (0.2)	2 (0.4)
Cord blood parasitaemia^†^	0 (0)	2 (0.4)

Numbers presented are n (%). Numbers may not add to sample size secondary to missing information.

^†^Parasitaemia was defined by presence of parasites on blood or tissue impression smear AND/OR by positive RDT. RDTs were not performed on cord blood.

* p < 0.05 comparing Rajnandgaon vs. Bastar districts.

**Table 4:** Univariate and multivariate logistic regression analysis of factors associated with peripheral parasitaemia and/or placental parasitaemia among pregnant women attending antenatal clinics and women at delivery units

	**ANC**			**DU**		
	**Univariate analysis**		**Multivariate analysis**	**Univariate analysis**		**Multivariate analysis**
**Potential factors**	**Peripheral parasitaemia prevalence**	**Odds ratio (95% CI)**	**Adjusted odds ratio (95% CI)**	**Peripheral parasitaemia prevalence**	**Odds ratio (95% CI)**	**Adjusted odds ratio (95% CI)**
District (malaria transmission)						
Stable (Bastar district)	34/1198 (2.8%)	43.7 (6.0-319.9)	43.4 (5.6-335.2)	18/474 (3.8%)	1.2 (0.6-2.4)	-
Unstable (Rajnandgoan district)	1/1498 (0.1%)			16/502 (3.2%)		
Age (years)						
< 20	5/242 (2.1%)	1.7 (0.7-4.4)	-	2/65 (3.1%)	0.9 (0.2-3.8)	-
≥ 20	30/2454 (1.2%)			31/909 (3.4%)		
Gravidity						
Primi-/secundigravid	26/2056 (1.3%)	0.9 (0.4-1.9)	-	28/768 (3.7%)	1.5 (0.6-4.0)	-
Multigravid	9/640 (1.4%)			5/206 (2.4%)		
Moderate anemia (< 9 g/dL)						
Yes	22/432 (5.1%)	9.3 (4.6-18.6)	3.7 (1.8 – 7.7)	11/145 (7.6%)	3.0 (1.4-6.3)	2.5 (1.1-5.4)
No	13/2261 (0.6%)			22/828 (2.7%)		
Fever						
Within past week	23/239 (9.6%)	21.7 (10.6-44.2)	3.2 (1.2 – 8.6)	8/50 (16.0%)	6.8 (2.9-16.1)	5.8 (2.4-13.9)
No fever in past week	12/2457 (0.5%)			25/923 (2.7%)		
Bednet use last night						
Yes	4/742 (0.5%)	0.9 (0.2-5.1)	-	5/203 (2.5%)	0.7 (0.3-1.8)	-
No	2/345 (0.6%)			28/771 (3.6%)		
Indoor residual spraying						
Yes (ever)	8/697 (1.2%)	0.8 (0.4-1.9)	-	4/261 (1.5%)	0.4 (0.1-1.1)	-
No (never)	27/1999 (1.4%)			29/712 (4.1%)		
Education						
No formal schooling	25/571 (4.4%)	7.8 (3.7-16.3)	4.6 (2.0 – 10.7)	13/265 (4.9%)	1.8 (0.9-3.6)	-
At least some schooling	10/1711 (0.6%)			20/709 (2.8%)		
Caste						
Tribal caste	15/602 (2.5%)	2.6 (1.3-5.2)	0.7 (0.3 -1.6)	10/282 (3.6%)	1.1 (0.5-2.3)	-
Other caste	20/2091 (1.0%)			23/690 (3.3%)		
Home ownership						
Yes	35/2348 (1.5%)	-		29/873 (3.3%)	0.8 (0.3-2.4)	-
No	0/348 (0%)			4/101 (4.0%)		
Wall material						
Mud	35/1942 (1.8%)	-		25/743 (3.4%)	1.0 (0.4-2.2)	-
No mud	0/754 (0%)			8/231 (3.5%)		
Primary cooking fuel						
Biomass	35/2134 (1.6%)	-		26/792 (3.3%)	0.8 (0.4-2.0)	-
Non-biomass	0/563 (0%)			7/181 (3.9%)		

**Table 5:** Prevalence of adverse singleton birth outcomes stratified by site and presence versus absence of placental parasitaemia

	**Rajnandgaon**		**Bastar**	
	**Placental parasitaemia**^¶^		**Placental parasitaemia**^¶^	
	**Present**	**Absent**	**Present**	**Absent**
	**n/d**^**†**^**(%)**	**n/d**^**†**^**(%)**	**n/d**^**†**^**(%)**	**n/d**^**†**^**(%)**
Stillbirth	0/15 (0)	12/487 (2.5)	1/16 (6.3)	10/448 (2.2)
Low birth weight	3/15 (20.0)	98/472 (20.8)	9/15 (60.0)^a^	128/437 (29.3)^a^
Gestational hypertension*	2/15 (13.3)	113/475 (23.8)	1/15 (6.7)	44/438 (10.1)
Birth weight (grams)	2500	2700	2280	2500
Median (range)	2050-3250	1400-3750	1700-3000^a^	1075-4085^a^
Gestational age (weeks)	38 (38-40)	38 (32-42)	36 (32-40)	36 (30-44)

^¶^ Parasitaemia defined by presence of parasites on blood or tissue impression smear and/or by positive rapid diagnostic test.

^†^Analysis is limited to singleton pregnancies where the placenta was examined for parasitaemia. For outcomes other than stillbirth, only live born infants were included.

**Figure 1:**
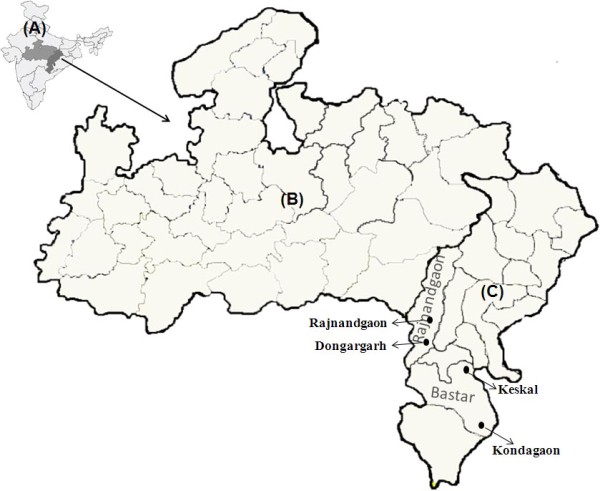
(A) Map of India (B) Madhya Pradesh (C) Chhattisgarh showing study area.

Malaria in pregnancy (MIP), especially when caused by *P. falciparum*, poses substantial risk to the mother and foetus by increasing the risk of foetal death, prematurity, low birth weight (LBW), and maternal anaemia [8]. These risks vary between stable and unstable settings.

Given the relatively limited data available on the burden of MIP in India, this study was undertaken in one high and one low transmission district in Chhattisgarh, a state with a large tribal population. The goal was to investigate the local epidemiology of malaria and its impact on pregnant women and their newborns. The specific objectives were to determine the prevalence of malaria during pregnancy in a population of pregnant women presenting for routine antenatal care, its association with maternal and birth outcomes, and use of anti-malarial preventive measures. Collectively, these data will help build an evidence base for development of policies to reduce the burden of MIP in central India.

## Methods

### Study site/design

This study was conducted in Chhattisgarh (formerly the eastern part of Madhya Pradesh), which was established on 1 November, 2000 as the 26th state of India (Figure 1). Situated in central India, Chhattisgarh contributes 9.5% of all annually reported malaria cases in India [9]. Scheduled tribes or indigenous people are a socio-economically deprived population in India. According to the 2001 Census, the scheduled tribe population of Chhattisgarh is 6,616,596 or 31.8% of the state population [10]. The geographical area of Chhattisgarh is 135,191 sq km, of which 55,772 sq km (41.25%) is forested. The climate is characterized by a hot summer (March to June), a monsoon/rainy season (July to October) and a cool autumn season (November to February).

This observational study consisted of cross-sectional surveys conducted over a period of 12 months at antenatal clinics (ANC), delivery units (DU) and female inpatient wards in two districts: Rajnandgaon and Bastar. Recruitment and enrolment took place from June 2007 to May 2008. In 2006 Rajnandgaon had unstable malaria transmission with a slide positivity rate (SPR) of 1.6%, while Bastar had stable seasonal transmission with a SPR of 12.8% [11]. In Rajnandgaon, 19.2% of malaria is due to *P. falciparum*. In Bastar, *P. falciparum* accounts for 89.8% of malaria [11]. All other episodes of malaria were due to *P. vivax*. High transmission areas are defined on the basis of persistently high slide positivity rate (SPR) and high *P. falciparum* percentage while low SPR and low *P. falciparum* percentage areas are classified as low or unstable transmission area. In recent years, annually two rounds of indoor residual spraying (IRS) with DDT were performed in Rajnandgaon. Two rounds of synthetic pyrethroid (alpha-cypermethrin) were used in Bastar as resistance to DDT has been noted in this district [11].

In each district a rural and an urban health facility were selected. The Keskal Community Health Center, the rural site in Bastar, performs approximately 750 ANC visits per year. In Rajnandgaon, the rural site was Dongargarh Community Health Center, which reports approximately 1,500 ANC visits annually. The urban sites were Kondagaon Ravindra Nath Taigore Community Health Center in Bastar district and the Rajnandgaon District Hospital, which average of 1,500 and 2,450 ANC visits per year, respectively. Most pregnant women in the two districts attended ANC for routine visits at least once during their pregnancy. In Rajnandgaon, 37% of pregnant women had one visit, 26% two visits, and 37% three or more visits. Similar figures for Bastar district included 32% of pregnant women attending one visit, 23% two visits, and 45% three or more visits. Both districts have reported more than 80% home deliveries [12]. As there is no specific policy for prevention of malaria during pregnancy in India, few pregnant women have insecticide-treated bed nets (ITNs) and chemoprophylaxis with chloroquine has not been implemented.

### Screening and enrolment

The study had three separate components recruiting distinct groups of women from ANCs, the delivery ward, and the inpatient ward. Pregnant women aged ≥15 years who presented to the ANC for routine care and were willing to provide written informed consent were enrolled. Those who had previously participated were excluded from participating again in the ANC component of the study but they could participate in the DU component. For the DU part of the study, women aged ≥15 years who presented for delivery and were willing to provide written informed consent were enrolled. During the study period at each of the health centres, any pregnant women admitted to the hospital with suspected malaria or associated complications were eligible for enrolment upon provision of written informed consent.

### Antenatal clinic procedures

Trained study personnel interviewed the enrolled women and collected information on sociodemographic characteristics, previous medical and obstetric history, current pregnancy complications, use of malarial prevention measures, fever, and malarial morbidity and treatment during the current pregnancy. A complete physical examination was performed including fundal height assessment and measurement of maternal height and weight. Height was measured with an anthropometric rod and a digital scale was used for measuring weight. This scale was accurate to 0.5 kg and the height rod to 1 mm. These scales were standardised weekly against known height and weight measurements. Axillary temperature was determined with a digital thermometer. Blood was obtained by finger-stick for haemoglobin determination, malaria rapid diagnostic test (RDT), and preparation of thin and thick blood smears. Women with positive RDT results or who were anaemic were referred immediately to the hospital physician for treatment. The study team also informed clinic staff when additional parasitaemic individuals were identified through blood smears so that treatment could be provided as per Indian national programme guidelines [13]. At the time of the study, treatment consisted of chloroquine for both *P. falciparum* and *P. vivax*. Iron and folic acid (60 mg iron + 400 μg folic acid daily) was given to all the pregnant women through the primary health care system under the national anaemia prophylaxis programme [14]. Data on nutritional status were not collected.

### Delivery unit procedures

Trained study personnel interviewed pregnant women enrolled at the DUs to collect similar information as described above under ANC procedures. A physical examination was performed and blood pressure was measured. Blood was obtained by maternal finger-stick soon after delivery for haemoglobin determination, RDT, thin and thick malaria blood film preparation. Placental blood was obtained by incision for preparation of thin and thick blood smears, and RDT. An impression smear from the maternal side of the placenta was also obtained. A drop of cord blood was taken to prepare blood smears; RDTs were not performed with cord blood. All neonates were weighed with an electronic digital scale to the nearest 10 gm and the gestational ages of all live births were estimated within 24 hours of delivery by means of a modified Ballard examination [15]. The digital scale used for weighing newborns was accurate to 0.005 kg. Women with positive RDT or blood smear results were referred for treatment. Details of delivery (including date and time of delivery, type, complications, and gestation) were abstracted from delivery records.

### Inpatient procedures

Enrolled subjects were interviewed and information on socio-economic status, reproductive history including obstetric history, history of fever and anti-malarial drug use, and use of anti-malarial prevention measures was collected. Data including recorded clinical signs, results of laboratory investigations, treatments administered, admission and discharge diagnosis and outcome of admission were extracted from the subject’s hospital record. Results of any blood smears performed in the hospital were reviewed, but study personnel did not perform additional smears.

### Laboratory procedures

Thick and thin smears prepared from peripheral blood of ANC and DU subjects, placental blood and placental impression from DU subjects, and cord blood were Giemsa-stained (3% stain for 45-60 minutes) and examined under 100× oil immersion [16]. Asexual parasite forms were counted until a minimum of 200 white blood cells (WBCs) had been examined. Slides were considered negative only if no parasites were seen after identifying 500 leukocytes. Parasite densities were estimated using an assumed total WBC count of 8,000 leukocytes/μL of blood [16]. The thin film was used to identify the *Plasmodium* species*.* All smears were re-checked by a member of the Parasitology Laboratory at the National Institute of Malaria Research (NIMR) Field Station in Jabalpur as a routine quality control measure. The First Response Malaria *Pf/Pv* test (PMC, Mumbai, India), a parasite lactic dehydrogenase antigen test, was used to perform the RDTs. This test has a sensitivity of 96% and specificity of 95% for detection of *P. falciparum* and a sensitivity of 83% and specificity of 94% for non-falciparum species in non-pregnant women [17]. A portable HemoCue machine (Ängelholm, Sweden), using a control with each assay, was used for haemoglobin determinations.

### Study definitions

Peripheral parasitaemia: presence of asexual *P. falciparum* or *P. vivax* parasitic forms on blood smears and/or positive RDT from peripheral blood. Placental parasitaemia: presence of asexual *P. falciparum* or *P. vivax* parasitic forms on placental blood or impression smear of maternal side of placenta or by RDT. Anaemia: haemoglobin <11 g/dL. Moderate/severe anaemia: haemoglobin <9 g/dL. Low birth weight: birth weight <2,500 g. Prematurity: gestational age <37 weeks as assessed by Ballard examination [15]. Stillbirth: death of a foetus before delivery in a pregnancy estimated at 28 weeks gestation through delivery.

### Ethical clearance

The Institutional Review Boards of Boston University and the Centers for Disease Control and Prevention, the Ethics Committee of the NIMR in India, the Scientific Advisory Committee of the NIMR, and the Health Ministry Screening Committee of the Indian Council of Medical Research (ICMR) reviewed and approved the study protocol and informed consent forms.

### Sample size

To estimate the sample size for a point estimate of the proportion of pregnant women with malaria parasitaemia, the following formula is used:$n=\frac{N{z_{\alpha /2}}^{2}p\left(1-p\right)}{d^{2}\left(N-1\right)+z_{\alpha /2}^{2}p\left(1-p\right)}$where N is the total population of the district being surveyed, d is the precision of the estimates, p is the estimated proportion of pregnant women with parasitaemia, and α is the type I error level. The sample size n was multiplied by an estimate of the design effect in order to correct for cluster sampling. For all of the sample size calculations, a large N (1,000,000), an α equal to 0.05, and a design effect of 2 was used. For the primary outcome “proportion of asymptomatic pregnant women with parasitaemia”, a target enrolment of 1,500 women in each study district was planned. Given a slide positivity rate for all *Plasmodium* species of approximately 2% in Rajnandgaon (unstable transmission), this sample size would give a 1% level of precision (2 ± 1%). In Bastar (stable transmission), with a slide positive rate of 13%, this allows approximately a 2.5% level of precision (13 ± 2.5%). Unless otherwise stated, the levels of precision that were calculated for each primary outcome are based on a composite of all *Plasmodium* infections.

### Data management and analysis

All case report forms were checked for completeness and inappropriate or illogical responses. The forms were double entered using CS-Pro (US Census Bureau, Washington, DC, USA), with range, consistency, and edit checks built into the data entry program for quality control. The two databases were validated and all inconsistencies and differences were resolved. Statistical analyses were performed using SAS software version 9.1 (SAS Institute, Cary, NC, USA). Categorical data are presented as frequency counts (percentages) and compared using the chi-square or Fisher’s exact statistic as appropriate. Continuous data are presented as mean ± standard deviation (SD) and compared using the t-test or analysis of variance as appropriate. Since most participants did not know their exact date of birth, participants’ ages in ranges based on their estimations are presented. For ANC participants, gestational age was calculated by fundal height when the fundus was above the umbilicus. Unrecorded fundal heights were considered a gestational age <20 weeks. Univariate logistic regression models were used to assess potential risk factors for parasitaemia. For ANC participants, the risk for peripheral parasitaemia and, for DU participants, the risk for either peripheral and/or placental parasitaemia was modelled. Odds ratios (OR) for identified factors associated with parasitaemia were then adjusted in multivariate models for other identified factors.

## Results

Of 2,698 pregnant women screened during their ANC visits, one was ineligible because she had previously participated in the study, one refused to provide informed consent, and 2,696 were enrolled. All 1,030 pregnant women screened in DU were eligible, though two refused to provide consent, and therefore 1,028 were enrolled. In addition, during the course of the 12-month study, the outcomes of 68 pregnant women admitted for malaria-related causes to the participating health facilities were reviewed.

### Baseline characteristics of antenatal clinic and delivery unit participants

Most pregnant women attending ANCs were 20-34 years old and in their 20th to 36th week of gestation (Table 1). In Bastar district, the site with stable malaria transmission, women were more likely to be primigravidae, have no formal schooling, and be from traditionally disadvantaged communities (scheduled tribes or scheduled castes). Pregnant women from Rajnandgaon were more likely to own a television whereas those from Bastar were more likely to own their house, although the latter homes were often constructed of mud bricks. The Bastar women also were more likely to use wood as their primary cooking fuel and less likely to use gas.

Most pregnant women enrolled at DUs in Bastar and Rajnandgaon were aged 20-34 years, but women in Bastar were more likely to be primigravidae, and had a lower mean gestational age at delivery than women in Rajnandgaon (Table 1). Similar to the ANC participants’ characteristics, women in Bastar were more likely to be from scheduled tribes or scheduled castes, to lack any formal schooling, and to own their own house. They were also less likely to own a television, to have a concrete house, or to cook with gas when compared to pregnant women attending DUs in Rajnandgaon. Overall these indicators suggest that participants in general from Bastar had lower socioeconomic status than those from Rajnandgaon.

### Use of malaria prevention measures

Less than half of women enrolled at either the ANCs or the DUs in both districts reported owning a bed net and very few households (<2%) reported that this was an ITN (Table 2). About 30% of hospitalized pregnant women reported owning a bed net in their household but none had an ITN. Among ANC subjects, bed net ownership was more common among pregnant women from Bastar compared with those from Rajnandgoan (45% *vs* 37%, p < 0.0001). Among ANC bed net owners, women in Bastar district were also more likely to have slept under the bed net the previous night and report sleeping under the net most nights. For DU participants, there was no significant difference in the proportion of bed nets in the household by district but bed net owners from Bastar were more likely to have slept under the net in the previous night and to report sleeping under a net most nights. Only seven women in the entire study, including both ANC and DU components, had received malaria prophylaxis and these women were all from Bastar. Only one of the seven women recalled the specific prophylactic medicine administered (sulphadoxine-pyrimethamine [SP]).

About a third of pregnant women in Rajnandgaon described having had IRS in their home in the last year, whereas about a fifth of households in Bastar had been sprayed.

### Prevalence of parasitaemia and anaemia

The overall prevalence of peripheral parasitaemia was relatively low among women presenting for antenatal care (1.3%, 95% CI 0.9 – 1.8; 35/2696) and among women at the time of delivery (1.9%, 95% CI 1.1 – 2.9; 19/1025) (Table 3). Peripheral parasitaemia was significantly more likely to be found among women from Bastar than among those from Rajnandgoan (p < 0.001 for both ANC and DU) (Table 3). *P. falciparum* was the predominant species identified in peripheral blood among women in Bastar (>80% of all infections), while *P. vivax* was the more frequent species among parasitaemic women in Rajnandgoan (75% of all positive cases).

Placental parasitaemia was also relatively rare among study participants but about twice as common as peripheral infections (3.4%, 95% CI 2.4 – 4.7; 33/978). There was no significant difference by district in placental parasitaemia although the species varied as it did among peripheral infections, with *P. vivax* encountered commonly in Rajnandgoan and *P. falciparum* more common in Bastar. Cord blood smears were positive in only two cases in Bastar, one with *P. falciparum* and the other with *P. vivax*.

The highest prevalence of peripheral and placental parasitaemia occurred from July to December in both ANC and DU participants. Among ANC participants, the density of parasitaemia varied widely (range: 120-296,640 asexual forms per μL; mean 24,993 ± 43,122 asexual forms per μL). Mean densities were higher among those infected with *P. falciparum* relative to those with *P. vivax* (26,899 *vs* 14,328 asexual forms per μL). Peripheral parasite density was lower among DU participants (range 80-15,120 asexual forms per μL; mean 2,402 ± 4,173 asexual forms per μL). Density of placental parasitaemia ranged from 80-12,480 asexual forms per μL (mean 2,381 ± 3,814).

Most subjects, including 67.5% of ANC and 74.6% of DU participants, were anaemic. Moderate to severe anaemia was less common, occurring in 16% and 15.1% of ANC and DU subjects, respectively. Moderate to severe anaemia was significantly more common among women from Bastar than those in Rajnandgoan in both ANCs and DUs (Table 3). Notably, 57% of pregnant women in Rajnandgaon and 62% in Bastar attending ANC reported that they took iron and folic acid supplements regularly. There was no assessment performed of adherence to these antenatal supplements nor was a dietary intake evaluation performed so it is not possible to determine whether poor adherence to supplements contributed to maternal anaemia.

### Factors associated with parasitaemia

The following potential risk factors for or predictors of parasitaemia were considered: (stable *vs* unstable malaria transmission), maternal age, gravidity, moderate anaemia, fever within past week, bed net use prior night, history of IRS in the home, education, caste, home ownership, wall material, and cooking fuel. For ANC participants, residence in Bastar district (stable malaria transmission) was strongly associated with peripheral parasitaemia (adjusted OR [aOR] 43.4; 95% CI, 5.6-335.2) (Table 4). Additional covariates associated with parasitaemia that remained significant after adjustment in multivariate analysis were moderate anaemia (aOR 3.7; 95% CI 1.8-7.7), fever within the past week (aOR 3.2; 95% CI 1.2-8.6), and lack of formal education (aOR 4.6; 95% CI 2.0-10.7).

Among DU participants, the only two significant covariates for peripheral and/or placental parasitaemia in multivariate analysis were moderate anaemia (aOR 2.5, 95% CI 1.1-5.4) and fever in the last week (aOR 5.8, 95% 2.4-13.9) (Table 4). Geographic residence in Bastar *vs* Rajnandgoan did not predict an increased risk of parasitaemia at delivery nor did gravidity.

### Adverse birth outcomes

Given the low parasitaemia prevalence among these study participants, particularly in Rajnandgoan district, it was difficult to fully explore the association of placental parasitaemia with adverse birth outcomes. With that limitation in mind, no association between placental parasitaemia and LBW, prematurity, or stillbirths was identified in Rajnandgaon (Table 5). By contrast, in Bastar district, placental parasitaemia was associated with an increased risk for LBW (60.0% *vs* 29.3%, p = 0.02) and a lower median birth weight (2,280 *vs* 2,500 gm, p = 0.001) but not prematurity.

### Inpatient admissions for malaria among pregnant women

There were 68 pregnant women admitted to the hospital for malaria-related causes during the 12-month study period including 26 in Rajnandgaon and 42 in Bastar. Overall, they represented 21.1% of pregnant women admitted for any medical treatment other than deliveries during the study period (10.9% of admissions in Rajnandgaon *vs* 50.6% in Bastar, p < 0.0001). When compared to Rajnandgaon, hospitalized pregnant women in Bastar were younger (mean age (years) 25 ± 4.3 *vs* 23 ± 3.7, p ≤ 0.05), more likely to be primigravidae (64.3% *vs* 34.6%, p < 0.025), more commonly from scheduled tribal caste (42.9% *vs* 15.4%, p < 0.025), and more likely to have had no formal schooling (p < 0.005). During admission, 82.3% of the women experienced a seizure (56/68) and more than half were severely anaemic (Hgb <7 g/dL). Admission and discharge diagnoses of malaria were more common in Bastar although only 52.4% (22/42) of the women in Bastar had laboratory-confirmed *P. falciparum* parasitaemia. None of the women in Rajnandgaon were parasitaemic nor were any hospitalized pregnant women in either district infected with *P. vivax* although diagnostic tests were not performed for all hospitalized women. There were no deaths among the malaria-related admissions.

## Discussion

The burden of malaria in pregnancy continues to be a public health challenge in parts of India. Each Indian state is unique with a variety of different ecosystems, disease transmission, and anti-malarial drug resistance. The most striking finding from this study conducted in central India in the state of Chhattisgarh was the confirmation that peripheral parasitaemia occurred more commonly in pregnant women presenting for antenatal care or for delivery in the high transmission district of Bastar compared to the low transmission district of Rajnandgaon. It was also evident that pregnant women in Bastar appeared to be at higher risk of hospitalization for malaria than those in Rajnandgaon. Moreover, placental infection was associated with LBW in Bastar district whereas the link with placental infection and birth outcomes was not evident in Rajnandgaon. Notably, there was a significant difference in the malaria species between districts with *P. falciparum* more common in Bastar district, potentially explaining that pregnant women with malaria in Bastar may be at higher risk for serious maternal complications and adverse birth outcomes.

The population of pregnant women enrolled from Bastar district differed from the population enrolled in Rajnandgaon with distinctions suggestive of an overall lower socioeconomic status and a higher proportion from traditionally disadvantaged communities (scheduled tribes or scheduled castes). The higher prevalence of malaria and its sequelae in pregnancy in Bastar district therefore may be due to associated low health literacy, lack of access to vector control methods, or limited access to anti-malarial drugs, or it may represent some local ecology unable to be identified in this study. Interestingly, IRS of the home, which is typically conducted by government, was reported more commonly in the low transmission district of Rajnandgaon. Bed net usage, which relies on the act of the individual, was more common in Bastar. These results highlight the potential need to enhance IRS in the high transmission district.

Several states in India, including Chhattisgarh, have benefitted from the Enhanced Malaria Control Project funded by the World Bank from 1997 to 2005, which emphasized an integrated approach to malaria control including early case detection and prompt treatment, vector control with targeted IRS, distribution of ITNs, increased public awareness through informational campaigns and strengthening of regional institutions to provide malaria surveillance and control of outbreaks. The EMCP used specific selection criteria to ensure a better focus on poor and inaccessible groups (such as areas with more than 25% tribal population) and areas with high malaria burden (annual parasite incidence >2 per thousand population and reported malaria-associated deaths). The EMCP mainly benefits the tribal population of the targeted districts of eight states and has the flexibility to divert resources to any needy areas in case of a malaria outbreak [18]. Bastar district was targeted for inclusion in EMCP efforts; however, as our results suggest, there remain continued opportunities for reducing the burden of malaria during pregnancy here. Although improving access to ITNs is a component of the EMCP, less than 1% of women owned ITNs. A little over a third of households owned untreated nets.

The two vectors active in the study area are *Anopheles culicifacies* and *Anopheles fluviatilis*, both of which are capable of transmitting both *P. falciparum* and *P. vivax*. *Anopheles culicifacies* is mainly endophilic and found in all seasons. This species is known to maintain malaria transmission from July to October [19] and accordingly, two rounds of IRS were recommended under the national programme for interruption of malaria transmission. By contrast, *An. fluviatilis* is mainly exophilic and found only during post monsoon and autumn seasons. Its role in malaria transmission is limited [20].

In addition to better establishing the prevalence of malaria in pregnancy in both a high and low transmission district, this study sought to identify risk factors associated with parasitaemia to inform evidence-based interventions to reduce the burden of malaria in pregnancy. As observed in population-based studies elsewhere in Asia [21], primigravidae and secundigravidae were not more likely to be parasitaemic than multigravidae. This suggests that efforts to reduce the burden of malaria in pregnancy should target all gravidae. These findings contrast with two studies elsewhere in India, which found that primi-and secundigravidae were at higher risk [22,23]. The multivariate analysis for both ANC and DU participants identified both fever within the prior week and moderate anaemia as predictors of parasitaemia. Neither is a true risk factor but rather a consequence of the malaria itself. While screening all pregnant women for malaria may be impractical and detract from other competing maternal-child health priorities, targeted screening of pregnant women presenting with fever is already being done as part of the national malaria control program. However, screening pregnant women with physical or laboratory evidence of anaemia using either traditional microscopy or RDTs should be considered since they appear to be at increased risk of malaria infection.

Pregnant women in Bastar were at higher risk of hospitalization for malaria than those in Rajnandgaon although there were no fatal outcomes. In contrast, Barnett *et al* recorded very high mortality (23%) due to malaria on the Jharkhand Orissa border using verbal autopsies [24]. Similarly a very high estimate of deaths (>200,000) due to malaria in the whole country was obtained by Dhingra *et al*[25]. However, the latter did not use slide or RDT confirmation for the deaths attributed to malaria so these results may have suffered from a misclassification bias. It is generally believed that there is some degree of uncertainty over assigning malaria as a cause of death without the availability of test results [24]. For example, verbal autopsy over estimated malaria deaths in children by 47% in a low transmission setting in Uganda [26] and by 200% in adults in a high transmission setting in Ghana where the proportion of mortality from malaria among adults was low [27]. Therefore, there is a need to generate accurate and reliable estimates by conducting community based surveys along with parasitologically-confirmed test results to overcome the uncertainty over assigning malaria as a cause of death.

A major limitation of this study was its focus on women attending government health facilities. This study did not evaluate women using private practitioners, those not receiving antenatal care, or those delivering in the community. An additional limitation of this study was the cross-sectional design. Cross-sectional surveys are prone to variation because they provide only an estimate of the prevalence of malaria infections in a short period. However, since this study was conducted over a 12-month period, it provides a more representative sample of the prevalence of malaria over all seasons. To better quantify the burden of malaria in pregnancy, longitudinal follow-up of pregnant women and their newborns would be preferable. The non-probabilistic sampling strategy used here (necessary due to limited resources) is another design weakness as this does not allow for generalization of the observations. Nevertheless, since this study was done over a full year period in two different districts and the majority of eligible women were willing to participate, the findings serve to present a reasonable picture of the actual malaria situation in Chhattisgarh. Another limitation was the dependence on hospital records for all data on the hospitalized pregnant women. Since this resulted in a substantial proportion not having specific malaria diagnostic tests, it is not possible to determine whether *P. vivax* contributed to the morbidity experienced by this cohort of hospitalized women.

Given the large proportion of malaria-associated illness among pregnant women hospitalized in Bastar district, improved efforts to reduce the burden of malaria in pregnancy in this poor area with large tribal populations are desperately needed. There should be a focus on increasing utilization of ANC services and improving case management of symptomatic pregnant women in primary care centres or the community to avoid the development of severe malaria requiring hospitalization. Since ITN availability was limited, there is a need to enhance ITN distribution and use. Further, since the retreatment of ITNs presents many logistical challenges, pregnant women should be given long-lasting, insecticide-treated nets (LLINs) at first ANC visits or through other social outreach programs as well as health education on prevention of MIP.

The low prevalence and the short seasonal predominance of transmission suggest prevention with intermittent preventive treatment (IPTp) with SP, as practiced in much of sub-Saharan Africa, might not be a feasible approach; presumptive use of IPTp throughout the year in this setting would result in unnecessary drug exposure in a large proportion of pregnant women. Furthermore, administration of intermittent seasonal IPTp in the peak transmission season would present major logistic challenges and would be complicated by the presence of both *P. vivax* and *P. falciparum*. Moreover, SP which is given for IPTp is not effective for *P. vivax*. The use of bivalent RDTs for point of care diagnosis of *P. falciparum* and *P. vivax*, and their prompt treatment with artemisinin-based combination therapy and chloroquine, respectively, to improve malaria case management for pregnant women represents a potentially better strategy [28]. Notably, an approach using intermittent screening and treatment of malaria resulted in similar levels of LBW in pregnant women of all gravidities in Ghana as IPTp [29].

## Conclusions

In areas with low levels of transmission or highly seasonal transmission, targeted screening, early diagnosis and subsequent treatment of symptomatic women during pregnancy might be more effective than a more widespread preventive approach [21,30]. However, in remote and inaccessible areas provision of services in government health facilities is limited, ANC attendance is inadequate, and compliance with therapy is poor. Since wide coverage of ANC services is not attainable, community-based health workers, such as *anganwadi* workers or village-based accredited social health activists (ASHA), could collect blood for RDTs/smears and deliver anti-malarial drugs [31]. This approach merits further study.

## Competing interests

The authors declare that they have no competing interests.

## Authors’ contributions

NS, BJW, KYA, CS, LS, MD, VU and DHH contributed to the conception and design of the study. MPS, MH and NS all contributed to study implementation and data collection. BJW and MPS performed data analyses and NS, KYA, LS, MD, VU and DHH assisted with interpretation of data. NS, BJW, LS, KYA and DHH drafted the manuscript. All authors contributed to and approved the final manuscript.

## References

[B1] Hay SI Okiro EA Gething PW Patil AP Tatem AJ Snow RW Estimating the global clinical burden of Plasmodium falciparum malaria in 2007 PLoS Med 2010 7 e1000290 10.1371/journal.pmed.1000290 20563310 PMC2885984

[B2] Guerra CA Howes RE Patil AP Gething PW Van Boeckel TP Temperley WH Kabaria CW Tatem AJ Manh BH Elyazar IR Baird JK Snow RW Hay SI The international limits and population at risk of Plasmodium vivax transmission in 2009 PLoS Negl Trop Dis 2010 4 e774 10.1371/journal.pntd.0000774 20689816 PMC2914753

[B3] Dhingra N Jha P Sharma VP Cohen AA Jotkar RM Rodriguez PS Bassani DG Suraweera W Million Death Study Collaborators: Adult and child malaria mortality in India: a nationally representative mortality survey Lancet 2010 376 1768 1774 10.1016/S0140-6736(10)60831-8 20970179 PMC3021416

[B4] WHO World Malaria Report 2009 World Health Organization, Geneva Available at: http://whqlibdoc.who.int/publications/2009/9789241563901_eng.PDF. (Accessed January 22, 2011)

[B5] Sharma VP Re-emergence of malaria in India Ind J Med Res 1996 103 26 45 8926025

[B6] Singh N Singh OP Sharma VP Dynamics of malaria transmission in forested and deforested region of Mandla district, Central India, Madhya Pradesh J Am Mosq Cont Assoc 1996 12 225 234 8827597

[B7] Sharma SK Tyagi PK Padhan K Upadhyay AK Haque MA Nanda N Joshi H Biswas S Adak T Das BS Chauhan VS Chitnis CE Subbarao SK Epidemiology of malaria transmission in forest and plain ecotype villages in Sundargarh District, Orissa, India Trans R Soc Trop Med Hyg 2006 100 917 925 10.1016/j.trstmh.2006.01.007 16697022

[B8] Desai M ter Kuile FO Nosten F McGready R Asamoa K Brabin B Newman RD Epidemiology and burden of malaria in pregnancy Lancet Infect Dis 2007 7 93 104 10.1016/S1473-3099(07)70021-X 17251080

[B9] National Vector Borne Disease Control Program Malaria situation in India Available at: http://nvbdcp.gov.in/Doc/Malaria-situation-Nov11.pdf. (Accessed January 22, 2012)

[B10] Census India Chhattisgarh data highlights: The schedule tribes. Census of India 2001 Available at: http://www.censusindia.gov.in/Tables_Published/SCST/dh_st_chhattisgarh.pdf. (Accessed January 22, 2012)

[B11] State Vector Borne Disease Control Program Malaria Control Program Annual Report, 2008-2010. State Vector Borne Disease Control Programme in Raipur, Chhattisgarh 2008-2010 Directorate of Health Services Chhattisgarh, India

[B12] District Level Household and Facility Survey (DLHS-3) 2007-08 2010 Chhattisgarh Available at: http://www.rchiips.org/pdf/rch3/report/ch.pdf. (Accessed July 20, 2012)

[B13] Guidelines for diagnosis and treatment of malaria 2010 Available at: http://nvbdcp.gov.in/Doc/Technical-Guidelines-Malaria-2010.pdf. (Accessed July 20, 2012)

[B14] Kalaivani K Prevalence and consequences of anaemia in pregnancy Indian J Med Res 2009 130 627 633 20090119

[B15] Ballard JL Khoury JC Wedig K Wang L Eilers-Walsman BL Lipp R New Ballard Score, expanded to include extremely premature infants J Pediatr 1991 119 417 423 10.1016/S0022-3476(05)82056-6 1880657

[B16] WHO Basic Malaria Microscopy, Part I. Learner's Guide 2010 2 World Health Organization, Geneva Available at: http://www.searo.who.int/LinkFiles/Malaria_malaria_microscopy_Learners_guide2010.pdf. (Accessed January 22, 2012)

[B17] Bharti PK Silawat N Singh PP Singh MP Shukla M Chand G Dash AP Singh N The usefulness of a new rapid diagnostic test, the First Response® Malaria Combo (pLDH/HRP2) card test, for malaria diagnosis in the forested belt of central India Malar J 2008 7 126 10.1186/1475-2875-7-126 18620560 PMC2478667

[B18] Dhingra N Joshi RD Dhillon GP Lal S Enhanced malaria control project for World Bank support under National Malaria Eradication Programme (NMEP) J Comm Dis 1997 29 201 208 9465524

[B19] Vaid BK Nagendra S Paithane PK Spring transmission of malaria due to Anopheles culicifacies in North Western Madhya Pradesh J Commun Dis 1974 6 270

[B20] Regional Medical Research Centre for Tribals Annual report 2009-10 2010 Available at: http://rmrct.org/files_rmrc_web/centre's_publications/Annual%20report/Annual%20%20Report%202009-10.pdf, (Accessed July 20, 2012)

[B21] Rijken MJ McGready R Boel ME Poespoprodjo R Singh N Syafruddin D Rogerson S Nosten F Malaria in pregnancy in the Asia-Pacific region Lancet Infect Dis 2012 12 75 88 10.1016/S1473-3099(11)70315-2 22192132

[B22] Hamer DH Singh MP Wylie BJ Yeboah-Antwi K Tuchman J Desai M Udhayakumar V Gupta P Brooks MI Shukla MM Awasthy K Sabin L MacLeod WB Dash AP Singh N Burden of malaria in pregnancy in Jharkhand State, India. Malar J 2009 8 210 10.1186/1475-2875-8-210 19728882 PMC2744702

[B23] Singh N Shukla MM Sharma VP Epidemiology of malaria in pregnancy Bull World Health Organ 1999 77 567 571 10444880 PMC2557706

[B24] Barnett S Nair N Tripathy P Borghi J Rath S Costello A A prospective key informant surveillance system to measure maternal mortality - findings from indigenous populations in Jharkhand and Orissa, India. BMC Pregnancy Childbirth 2008 28 6 18307796 10.1186/1471-2393-8-6 PMC2268911

[B25] Dhingra N Jha P Sharma VP Cohen AA Jotkar RM Rodriguez PS Bassani DG Suraweera W Laxminarayan R Peto R Million Death Study Collaborators: Adult and child malaria mortality in India: a nationally representative mortality survey Lancet 2010 376 1768 1774 10.1016/S0140-6736(10)60831-8 20970179 PMC3021416

[B26] Snow RW Armstrong JR Forster D Winstanley MT Marsh VM Newton CR Waruiru C Mwangi I Winstanley PA Marsh K Childhood deaths in Africa: uses and limitations of verbal autopsies Lancet 1992 340 351 355 10.1016/0140-6736(92)91414-4 1353814

[B27] Chandramohan D Maude GH Rodrigues LC Hayes RJ Verbal autopsies for adult deaths: their development and validation in a multicentre study Trop Med Int Health 1998 3 436 446 10.1046/j.1365-3156.1998.00255.x 9657505

[B28] Singh N Shukla MM Shukla MK Mehra RK Sharma S Bharti PK Singh MP Singh A Gunasekar A Field and laboratory comparative evaluation of rapid malaria diagnostic tests versus traditional and molecular techniques in India Malar J 2010 9 191 10.1186/1475-2875-9-191 20602766 PMC2905433

[B29] Tagbor H Bruce J Agbo M Greenwood B Chandramohan D Intermittent screening and treatment versus intermittent preventive treatment of malaria in pregnancy: a randomised controlled non-inferiority trial PLoS ONE 2010 5 e14425 10.1371/journal.pone.0014425 21203389 PMC3010999

[B30] McGready R Davison BB Stepniewska K Cho T Shee H Brockman A Udomsangpetch R Looareesuwan S White NJ Meshnick SR Nosten F The effects of Plasmodium falciparum and P. vivax infections on placental histopathology in an area of low malaria transmission Am J Trop Med Hyg 2004 70 398 407 15100454

[B31] NRHM Report of the 2nd Common Review Mission 2008 Chhattisgarh 16-22 December 2008. Available at: http://nhsrcindia.org/download.php?downloadname=pdf_files/resources_thematic/Health_Sector_Overview/NHSRC_Contribution/179.pdf, (Accessed April 25, 2011)

